# Effects of Water-Soluble and Fat-Soluble Antioxidant Combinations in Oil-in-Water Emulsions on the Oxidative Stability of Walnut Kernels

**DOI:** 10.3390/foods14111967

**Published:** 2025-05-31

**Authors:** Ying Jing, Rongrong Wang, Huiliang Wen, Jianhua Xie

**Affiliations:** State Key Laboratory of Food Science and Resources, Nanchang University, Nanchang 330047, China; ncujingying@email.ncu.edu.cn (Y.J.); wangrongr4@163.com (R.W.)

**Keywords:** walnut, antioxidant, oxidative stability, storage

## Abstract

Walnuts, which are rich in unsaturated fatty acids (UFAs), are highly susceptible to oxidation during storage, leading to quality degradation. Consequently, antioxidant technologies for the oxidative stability of walnuts have garnered significant attention. The addition of antioxidants remains the most cost-effective and efficient method currently available, with synergistic effects enhancing the efficacy of mixed antioxidant combinations compared to single antioxidants. In this study, four lipophilic antioxidants—tert-butylhydroquinone (TBHQ), butylated hydroxytoluene (BHT), dilauryl thiodipropionate (DLTP), and propyl gallate (PG)—were paired with four hydrophilic antioxidants—rosemary extract (RE), phytic acid (PA), tea polyphenols (TPs), and sodium ascorbate (SA)—resulting in 16 experimental groups to investigate synergistic effects. The effects of water-soluble and fat-soluble antioxidant combinations on walnut oxidation were systematically evaluated through peroxide value, acid value, thiobarbituric acid reactive substances, and DPPH radical scavenging capacities. Additionally, fatty acid composition analysis was employed to assess the preservation of beneficial UFAs. Mechanistic insights were obtained via thermogravimetric analysis and electron paramagnetic resonance spectroscopy. Notably, two combinations, 0.03% TBHQ + 0.03% TPs (*w*/*w*) and 0.03% DLTP + 0.03% SA (*w*/*w*), exhibited good oxidative stability of walnut kernels. These formulations demonstrated superior antioxidant performance and effectively inhibited oxidative pathways while maintaining UFA integrity, demonstrating their potential as advanced preservation strategies for lipid-rich foods.

## 1. Introduction

Walnut (*Juglans regia* L.) is the seed of the most widespread distributed tree within the walnut family (*Juglandaceae*) [1,2]. It is one of the most common nuts and an important woody oilseed [3], with the oil content in its kernels ranging from 60 to 72% [4]. Over 90% of total fatty acids are unsaturated fatty acids (UFAs), which play significant roles in preventing cardiovascular diseases, reducing blood lipids, and promoting brain and retinal development in infants [5,6,7], particularly linoleic acid and α-linolenic acid. Additionally, walnuts contain trace nutrients [8] and bioactive compounds such as tocopherols, polyphenols, and phytosterols [9]. In summary, walnuts, as a high-quality oil-bearing nut, offer substantial health benefits to humans [10].

Walnut oil is rich in UFAs, primarily oleic acid, linoleic acid, and α-linolenic acid [11]. These UFAs, particularly linoleic and α-linolenic acids, can be metabolized into eicosapentaenoic acid (EPA) and docosahexaenoic acid (DHA), which exert health-promoting effects on humans [12,13]. Nevertheless, UFAs are highly susceptible to oxidation and lipolysis during high-temperature processing and storage [14]. This leads to changes in fatty acid composition, the formation of oxidation products and free radicals, loss of nutritional components, and degradation of sensory quality in oils. Therefore, measures to inhibit or delay walnut oil oxidation are essential. To address this, researchers have developed multiple antioxidant strategies, which can be broadly categorized into physical and chemical approaches. Physical methods include low-temperature storage [15] and controlled atmosphere storage [16], while chemical interventions involve the addition of synthetic or natural antioxidants [17,18]. Additionally, emerging technologies such as radiation treatment have shown potential in mitigating oxidative degradation [19,20].

Among these, the addition of antioxidants remains the most effective and cost-efficient method currently. Antioxidants can delay the oxidation, decomposition, and spoilage of oils. They can be categorized based on their source into natural and synthetic antioxidants, and based on their solubility into fat-soluble and water-soluble antioxidants. The antioxidant system comprises three distinct categories based on solubility characteristics: (1) primary lipid-soluble antioxidants including butylated hydroxytoluene (BHT), tert-butylhydroquinone (TBHQ), dilauryl thiodipropionate (DLTP), and propyl gallate (PG); (2) water-soluble antioxidants such as tea polyphenols (TPs), phytic acid (PA), sodium ascorbate (SA), and rosemary extract (RE); and (3) amphipathic antioxidants represented by ascorbic palmitate (AP). Given their diverse chemical structures, these antioxidants exhibit distinct mechanisms of action [21]. Existing research has identified both antagonistic and synergistic interactions among different antioxidants. To enhance the efficacy of antioxidant systems, further investigation into the synergistic effects of various antioxidants is essential.

Previous studies have demonstrated that combinations of binary or ternary antioxidants are more effective than single antioxidants at the same dose [22]. For instance, Tang et al. [23] reported that tocopherols and phospholipids exhibit synergistic effects on the oxidative stability of peony seed oil. Similarly, a combination of four types of antioxidants—tocopherol, ascorbyl palmitate, phytic acid, and polyphenol source (bamboo leaf, rosemary extract, tea polyphenols extract and polyphenol tea palmitate)—has been shown to enhance the oxidative stability of flaxseed oil [24]. Despite these findings, research on the combination of water-soluble and fat-soluble antioxidants in walnut kernels remains limited. Water-soluble antioxidants such as phytic acid and sodium ascorbate are nearly insoluble in the oil phase, while fat-soluble antioxidants like BHT have extremely low solubility in the water phase. This limits the synergistic potential of different solubility antioxidants in walnuts. We found that emulsifiers can overcome this limitation by reducing interfacial tension between the oil and water phases. Specifically, the addition of monoglyceride as an emulsifier can form a relatively stable emulsion system, thereby enhancing the compatibility of the oil and water phases [25].

The combination of α-tocopherol and green tea extract exhibited significant synergistic effects in the linoleic acid methyl ester emulsion system and sunflower seed oil, whereas only additive effects were observed in liposomes [26]. Factors influencing the synergistic effects between antioxidant combinations include concentration ratios [27], humidity [28], temperature [29], and light intensity [30].

Conversely, some complex antioxidant combinations may exhibit antagonistic effects under unfavorable conditions. For instance, α-tocopherol and quercetin demonstrated synergistic antioxidant effects in methyl linoleate emulsions and liposome systems, which may arise from interfacial binding of quercetin at oil/water boundaries protecting α-tocopherol against metmyoglobin-generated radicals in the aqueous phase, whereas antagonistic effects were observed in sunflower seed oil [31]. Similarly, rutin and tocotrienol in linoleic acid emulsions at concentrations ranging from 5 to 10 μm exhibited antagonistic effects, likely due to hydrogen bonding between rutin and tocotrienol [32]. In brief, the synergistic effects between antioxidants have emerged as a key research focus in the study of oil antioxidant properties. The development and application of antioxidant combinations are becoming increasingly prevalent in the food industry, moving beyond single antioxidants. Thus, developing synergistic binary antioxidant combinations can significantly enhance the effectiveness of antioxidants in walnuts.

This study simulated industrial processing techniques by employing synergistic binary antioxidant combinations to treat walnut kernels. The oxidation of walnut oil was characterized by measuring the peroxide value (POV), acid value (AV), thiobarbituric acid reactive substances (TBARSs), DPPH radical scavenging ability (DPPH), fatty acid composition (FA), thermogravimetric analysis (TG), and electron spin resonance (ESR) spectroscopy. The study explored the antioxidant effects of water-soluble (RE, TPs, PA, and SA) and fat-soluble (TBHQ, BHT, PG, and DLTP) antioxidants in oil-in-water emulsions. It compared the oxidative stability of oils treated with different antioxidants to identify new binary combinations that are more effective than commercial antioxidant blends and better suited for walnut preservation.

## 2. Materials and Methods

### 2.1. Materials and Reagents

Thin-skinned walnut kernels from Aksu City (Xinjiang, China) were procured from Fenyang Yuankang Special Products Co., Ltd. (Fenyang, Shanxi, China). Antioxidants including TBHQ, DLTP, PG, BHT, PA, RE, SA, and glycerol monostearate were supplied by Suihong Biotechnology Co., Ltd. (Yichun, Jiangxi, China). Tea polyphenols (TPs) were provided by Zenong Tea Chemical Technology Co., Ltd. (Shangrao, Jiangxi, China), while the commercial compound antioxidant (positive control, trade name: Composite Antioxidant for Nut Roasting-A Type) containing tert-butylhydroquinone (TBHQ), as the primary active component with auxiliary excipients, was sourced from Anhui Tianlitai Food Technology Development Co., Ltd. (Hefei, Anhui, China). Corn oil was acquired from Tianhong Supermarket (Nanchang, Jiangxi, China). DPPH (1,1-diphenyl-2-picrylhydrazine) and hydrogen peroxide (30%) were purchased from TCI Chemical Industry Development Co., Ltd. (Shanghai, China) and Hubble Chemical Technology Ltd. (Shanghai, China), respectively. Chromatographic-grade n-hexane was provided by Macklin Biochemical Technology Co., Ltd. (Shanghai, China). The standards for fatty acid determination were purchased from Nu-Chek Prep (Elysian, MN, USA). Other analytical-grade reagents were obtained from Aladdin Biochemical Technology Co., Ltd. (Shanghai, China).

### 2.2. Preparation of O/W Antioxidant Emulsion and Coating of Walnut Kernels with Antioxidants

Preparation of O/W antioxidant emulsion. The O/W emulsion was prepared by mixing corn oil and water at a ratio of 3:97 (*w*/*w*). Glycerol monostearate was incorporated as an emulsifier at a concentration of 0.25% (*w*/*w*) based on the total mass of the O/W emulsion. Then, a high-speed homogenizing emulsifier (RCD-1A, FLUKO Equipment Co., Ltd., Shanghai, China) was used to prepare the crude emulsion at 12,000 rpm for 4 min under room temperature conditions (25 ± 2 °C).

Antioxidant treatment of walnut kernels. Walnut kernels with uniform color, fullness, size, surface area, and freshness were selected. According to the protocol of Ma et al. [33], two antioxidants were mixed, and the addition amount was 1/2 of the maximum allowable dosage for each antioxidant. Subsequently, the antioxidant combinations were dissolved in the O/W emulsion at the concentrations specified in Table 1. The blank control group received immersion in antioxidant-free O/W emulsion, while the positive control group utilized commercial compound antioxidants incorporated at 0.06% (*w*/*w*) relative to emulsion weight. All emulsion systems (with/without antioxidants) underwent preheating to 60 °C prior to 10-min kernel immersion under strictly maintained 1:3 (*w*/*v*) walnut-to-emulsion proportioning. The walnut kernels were then removed and drained.

### 2.3. Accelerated Oxidation Treatment and Extraction of Walnut Oil

The selection of 60 °C for accelerated oxidation treatment was based on the AOCS Cg 5–97 Recommended Practice. Walnut kernels were placed in a 60 °C oven for accelerated oxidation treatment. Samples were collected on days 4, 7, and 9. The positions of the samples were regularly rotated to minimize positional effects within the oven. Walnut oil was extracted following a modified method based on Zhong et al. [34]. Walnut kernels were crushed using a high-speed grinder. The walnut powder was mixed with twice the volume of petroleum ether, shaken for 1 h, and left in the dark for 12 h to facilitate extraction. The mixture was filtered through a Buchner funnel, and the filtrate was evaporated using rotary distillation to remove the petroleum ether, yielding crude oil. The crude oil was then centrifuged at 3863× *g* for 10 min to remove water and impurities. Walnut oil containing various antioxidant combinations was collected for further analysis.

### 2.4. Determination of Peroxide Value

The determination of peroxide value (POV) was based on the method of Zhang et al. [35]. Specifically, 0.5 g walnut oil was diluted to 10 mL with a chloroform–methanol mixture (7:3, *v*/*v*). A 50 μL aliquot of a 3.5 g/L ferrous chloride solution was added to 1 mL of the s diluted sample, which was then further diluted to 10 mL with the chloroform–methanol mixture. After thorough mixing, 50 μL of a 300 g/L potassium thiocyanate solution was added. The absorbance of the resulting solution was measured by a dual-beam UV-visible spectrophotometer (TU-1900, Beijing Persee General Instrument Co., Ltd., Beijing, China) at 500 nm after 5 min. The POV was calculated through the calibration curve, and was calculated according to the following formula:

(1) $$POV=\left(C-C_{0}\right)/\left[m\times \left(V_{2}/V_{1}\right)\times 55.84\times 2\right]$$

where POV represents the peroxide value (meq/kg), C is the mass of iron in the sample determined using a calibration curve (μg), C_0_ is the mass of iron in the blank (μg), V_1_ is the dilution volumes of the oil sample (mL), V_2_ is the volume of sample (mL), m is the mass of the oil sample (g), 55.84 is the atomic weight of iron, and 2 is the conversion factor.

### 2.5. Determination of Acid Value

The determination of acid value (AV) was based on the method of Cho et al. [36] with appropriate modifications. Briefly, 3 g of walnut oil was weighed and mixed with 50 mL of petroleum ether–ethanol solution (1:1, *v*/*v*). After thorough mixing, 150 μL of a pheno–phthalein indicator solution (10 mg/mL in 95% ethanol) was added. Titration was performed using a 0.1 M potassium hydroxide ethanol solution, with the volume consumed being recorded. The titration endpoint was reached when the solution initially turned slightly pink and remained so for at least 15 s without significant fading. The AV was determined using the following formula:

(2) AV=56.11Vc/m

where AV is the acid value which is calculated by potassium hydroxide (mg/kg), V is the volume of potassium hydroxide standard titration solution consumed (mL), c is the actual concentration of potassium hydroxide standard titration (mol/L), m is the mass of oil sample (g), and 56.11 is the molar mass of potassium hydroxide (g/mol).

### 2.6. Determination of TBARS

The malondialdehyde (MDA) content was determined using the thiobarbituric acid reactive substance (TBARS) assay described by Li et al. [37] with appropriate modifications. Briefly, 150 mg of walnut oil was mixed with 1-butanol and diluted to a total volume of 25 mL. A 5 mL aliquot of this solution was mixed with 5 mL of a 2 mg/mL thiobarbituric acid solution, vortexed thoroughly, and heated in a water bath at 95 °C for 2 h. After reaching room temperature, the absorbance was measured by a dual-beam UV-visible spectrophotometer at 530 nm, with distilled water serving as the reference. The blank control group was prepared by replacing the oil sample with 1-butanol. The TBARS value was determined using the following formula:

(3) $$TBARS=50\left(A-B\right)/m$$

where TBARS is the content of malondialdehyde (mg/kg), A is the absorbance of the sample, B is the absorbance of the blank control, m is the mass of oil (mg), and 50 is effective factor.

### 2.7. Determination of DPPH Radical Scavenging Ability

The determination of DPPH radical scavenging ability was based on the method of Xu et al. [38] with appropriate modifications. The oil was first diluted to a concentration of 10 mg/mL in anhydrous ethanol. Subsequently, 4 mL of a 0.04 mg/mL DPPH solution was added to 4 mL of the sample solution and vortexed thoroughly. After incubating at room temperature under dark conditions for 30 min, the absorbance was measured by a spectrophotometer at 517 nm, with distilled water as the blank control. The DPPH radical scavenging ability of samples was determined using the following formula:

(4) $$DPPH=\left[1-\left(A_{1}-A_{2}\right)/A_{3}\right]\times 100\%$$

where A_1_ is the absorbance of sample and DPPH solution, A_2_ is the absorbance of sample and anhydrous ethanol, and A_3_ is the absorbance of anhydrous ethanol and DPPH solution.

### 2.8. Fatty Acid Composition

The determination of fatty acids was conducted using gas chromatography based on the method of Xu et al. [39]. Briefly, 10.00 mg of the oil sample was weighed, and 10 μL of C21:0 internal standard solution (5 mg/mL), 2 mL of chromatography grade n-hexane, and 100 μL potassium hydroxide methanol solution (2 mol/L) were added and mixed. The mixture was vortex-mixed for 30 s, followed by centrifugation at 2173× *g* for 5 min. The top layer was filtered through a 0.22 µm organic phase nylon needle filter for subsequent analysis.

An Agilent 8890 gas chromatograph (Agilent Technologies Inc., Santa Clara, CA, USA) equipped with a flame ionization detector (FID), a CP-Sil 88 type capillary chromatography column (100 m × 0.25 mm × 0.39 mm, 0.2 μm; Varian Inc., Palo Alto, CA, USA), and an auto sampler, was used to separate and quantify the FAMEs (fatty acid methyl esters). The oven temperature program was as follows: 60 °C for 5 min, then increased to 170 °C at a rate of 11.5 °C/min, held at 170 °C for 25 min, increased to 200 °C at 5 °C/min, held at 200 °C for 5 min, increased to 215 °C at 2 °C/min, and finally held at 215 °C for 20 min. The injection volume was 1 μL, with an injector temperature of 250 °C and a detector temperature of 250 °C. Hydrogen was used as the carrier gas at a velocity of 26 cm/s, with a split ratio of 10:1. The air flow was set at 300 mL/min, and the makeup gas flow was 20 mL/min.

### 2.9. Thermogravimetric Analysis

Thermogravimetric analysis (TG) was performed using a thermogravimetric analyzer (TGA-4000, PerkinElmer, Waltham, MA, USA) to continuously measure the weight of a sample under high-temperature conditions, providing insights into thermal stability and oxidation behavior [40]. An amount of 5 mg of walnut oil was weighed and placed in a sealed aluminum concave plate with a hole in the middle. Program heating was conducted with an initial temperature of 30 °C and a heating rate of 10 °C/min to increase to 500 °C. Nitrogen gas is the blowing gas to test the oxidative stability of walnut oil.

### 2.10. Detection of Intermediary Radicals by ESR Spectroscopy

The free radical signal intensity of walnut oil samples was monitored using electron spin resonance (ESR), following the methods described by Chen et al. [41] and Shen et al. [42]. The spin capture method based on 5,5-dimethyl-1-pyrroline-N-oxide (DMPO) as the capture agent is commonly used for determining the generation of oil oxygen radicals during thermal oxidation [43]. A 3% DMPO capturing agent solution was prepared by dissolving 30 mg of DMPO in 1 mL of toluene, avoiding light exposure during preparation and storage at −20 °C. For the experiment, 500 μL of walnut oil and 50 μL of the DMPO solution were mixed in a pressure tube, vortexed, and heated in a sand bath at 165 °C for 20 min. The sample was then transferred into a capillary tube, sealed with solid glue, and immediately placed in a high-sensitivity resonator. After baseline correction, the ESR instrument was used to measure the intensity of the free radical signals. The ESR parameters were set as follows: center field strength 3420 G, scanning width 100 G, scanning time 13 s, scanning frequency 10 times, and modulation amplitude 1.000 G.

### 2.11. Statistical Analysis

All experiments were performed in triplicate and data were expressed as means ± standard deviation (SD). Statistical analysis was performed with SPSS software (version 26.0, IBM Corp. Armonk, NY, USA), and *p* < 0.05 was considered statistically significant.

## 3. Results

### 3.1. Peroxide Value

**Figure 1:**
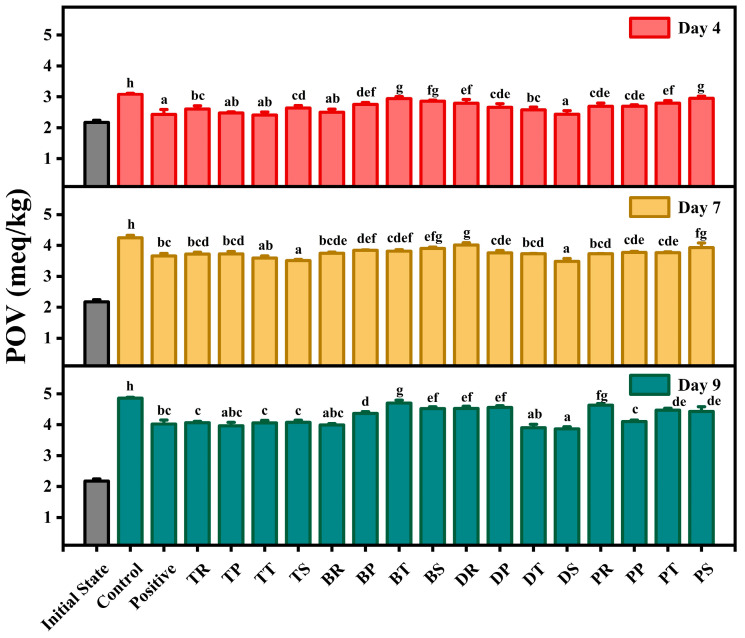
The effect of different antioxidant combinations on the peroxide value of walnuts during accelerated oxidation. There is no significant difference between groups represented by the same letters (*p* < 0.05).

POV is a measure of the hydroperoxide content in oils and is a key indicator for assessing their initial quality and storage stability. Higher POVs typically indicate poorer oil quality [44]. Figure 1 illustrates the impact of various antioxidant combinations on the POV of walnut oil during accelerated oxidation. The control group contained no antioxidants, whereas the positive control included a commercial compound antioxidant. Initially, the lowest POV was observed, reflecting minimal oxidation. As oxidation progressed, the POV increased due to the formation of hydroperoxides, which are primary oxidation products of UFAs in the presence of oxygen [45]. Compared to the control, the addition of antioxidants effectively reduced the peroxide value (POV) and delayed the oxidation of walnut oil. Relative to the positive control (commercial compound antioxidant), TT and DS demonstrated superior antioxidant effects. On day 4, TT exhibited the lowest POV, with no significant difference compared to the positive control, DS, TP, and BR, indicating that these groups equally delayed primary oxidation and performed well. On day 7, DS, TS, and TT maintained lower POV than the positive control, highlighting their significant ability to delay hydroperoxide formation and their robust antioxidant capacity. By day 9, DS, DT, TP, and BR all showed lower POV than the positive control, demonstrating a significant capacity to delay hydroperoxide formation in walnut oil. Overall, DS consistently exhibited strong antioxidant capacity across all three stages of accelerated oxidation. This can be attributed to the complementary mechanisms of its binary components: DLTP acts as a hydroperoxide decomposer to break down primary oxidation products, while SA scavenges aqueous-phase radicals and regenerates oxidized DLTP through electron transfer, establishing a redox cycle that sustains interfacial protection, thereby prolonging oxidative stability. TT effectively inhibited primary oxidation within the first 7 days but showed limited capacity in the later stages, with its antioxidant effect falling below that of the positive control by day 9. This may be attributed to the inherent properties of the specific antioxidants used. Additionally, TP, DT, and BR demonstrated antioxidant capacity in the later stages of oxidation. These antioxidant combinations showed effects comparable to the positive control, suggesting potential as alternatives to commercial compound antioxidants.

DLTP can serve as a peroxide decomposition agent for antioxidation [46]. Consequently, the combinations of DLTP with sodium ascorbate (DLTP-SA) and DLTP with tea polyphenols (DLTP-TPs) demonstrated robust antioxidant capacities in this study. [47] found that the combined effect of TPs+TBHQ is superior to their individual effects. The strong antioxidant capacity of TBHQ and TPs is likely due to the regenerative effect of TPs on TBHQ, a conclusion supported by Guo et al. [48].

### 3.2. Acid Value

**Figure 2:**
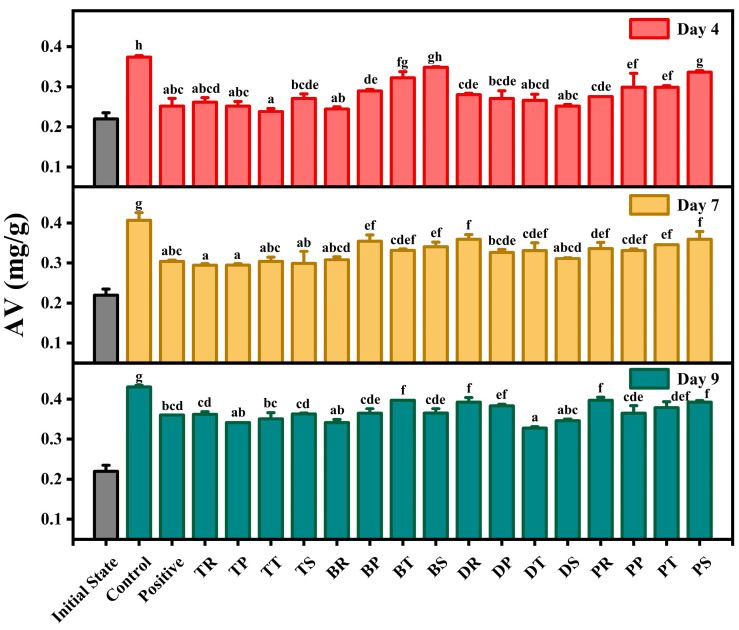
The effect of different antioxidant combinations on the acid value of walnuts during accelerated oxidation. There is no significant difference between groups represented by the same letters (*p* < 0.05).

AV represents the content of free fatty acids in oils and is commonly used to characterize the freshness of oil [49]. Walnut oil with lower AV exhibits better oxidative stability and is more suitable for human consumption [50]. Figure 2 shows the impact of various antioxidant combinations on the AV of walnut oil during oxidation. Initially, the AV was at its lowest. Overall, AV increased gradually as oxidation progressed. This indicates that walnut oil hydrolysis occurred during oxidation, leading to an increase in free fatty acids. The AV of walnut oil treated with antioxidant combinations was lower than that of the control, suggesting that antioxidants inhibited free fatty acid formation and mitigated oxidation. On day 4, compared to the positive control, TT and BR exhibited lower AV levels, while DS, TP, TR, and DT were slightly higher. By day 7, TR, TP, TT, and TS showed better antioxidant effects than the positive control, though not significantly. This suggests that TBHQ played a significant role at this stage. On day 9, DT, TP, BR, and DS demonstrated significantly better antioxidant effects than the positive control, while TT was higher but not significantly different. Across all three stages of oxidation, TT and BR exhibited stable antioxidant capacity, followed by DT, TP, and DS.

### 3.3. TBARS

**Figure 3:**
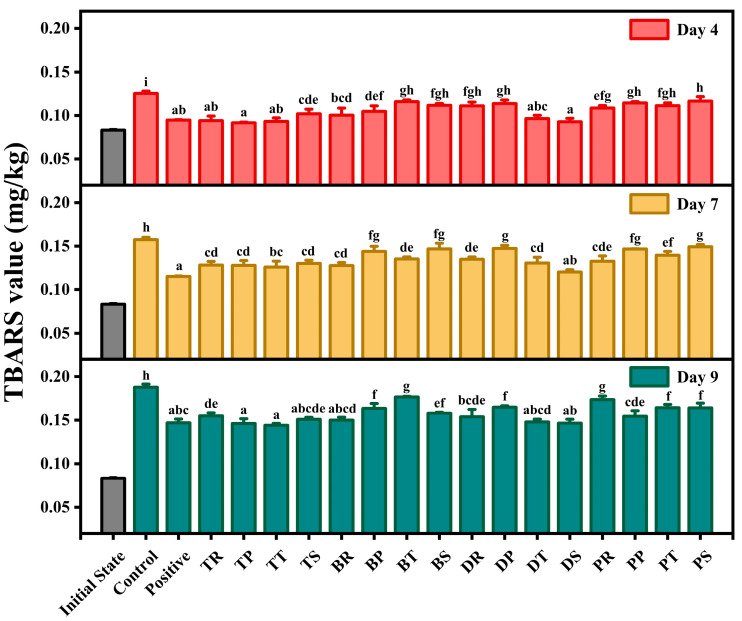
The effect of different antioxidant combinations on the TBARS value of walnuts during accelerated oxidation. There is no significant difference between groups represented by the same letters (*p* < 0.05).

UFAs in oils initially oxidize to form hydroperoxides, which subsequently decompose into carbonyl compounds through oxidative processes [51]. This sequence of reactions can impart an unpleasant odor to walnut oil. The TBARS value, representing the concentration of malondialdehyde (MDA) in 1 kg of oil, is a common metric for assessing secondary oxidation [52]. Lower TBARS values indicate better oxidative stability and less rancidity in walnut oil. Figure 3 illustrates the impact of various antioxidant combinations on the TBARS values of oxidized walnut oil. As oxidation time increased, TBARS values rose, indicating further oxidation of walnut oil. Compared to the control, antioxidants significantly reduced TBARS values and slowed oxidation. On day 4, TP, DS, TT, and TR exhibited lower TBARS values than the positive control, with no significant differences, suggesting they effectively delayed secondary oxidation. By day 7, only DS showed similar antioxidant effects to the positive control, while other groups had significantly lower TBARS values. On day 9, TT, TP, and DS maintained lower TBARS values than the positive control, whereas DT, BR, and TS had higher values but showed no significant differences from the positive control. Combining the results of POV and AV, it is evident that DS and TT significantly delayed both primary and secondary oxidation processes throughout the accelerated oxidation period. TP, DT, and BR demonstrated strong inhibitory effects on the formation of hydroperoxides and MDA in the later stages of oxidation, confirming their robust antioxidant capacity.

### 3.4. DPPH Radical Scavenging Ability

The DPPH radical scavenging ability of antioxidant combinations was assessed by monitoring the color change in sample solutions mixed with DPPH using a UV-visible spectrophotometer. Lower absorbance values indicate stronger scavenging abilities [45]. The proposed mechanism involves the activation of one hydroxyl group in the antioxidant molecule by another heterologous hydroxyl group, enhancing the formation of hydrogen protons that rapidly react with DPPH radicals [53].

**Figure 4:**
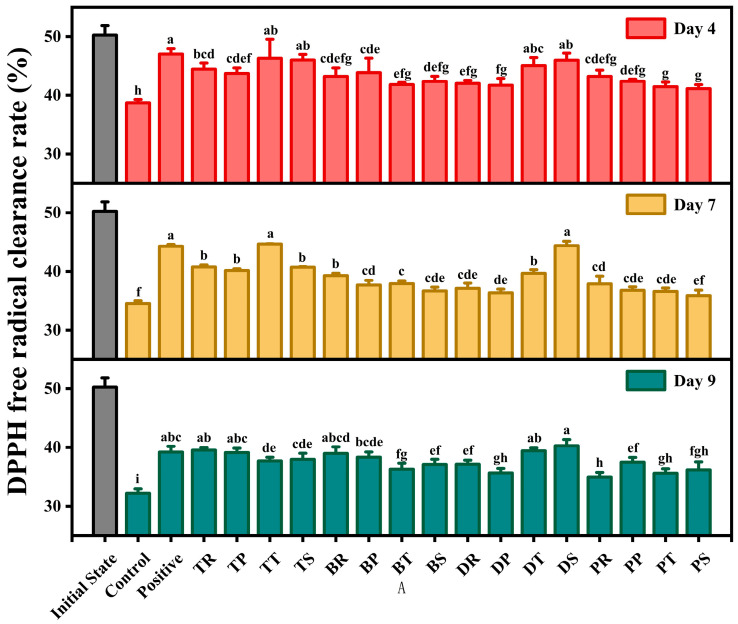
The effect of different antioxidant combinations on the DPPH free radical clearance rate of walnuts during accelerated oxidation. There is no significant difference between groups represented by the same letters (*p* < 0.05).

Overall, the DPPH free radical scavenging ability of antioxidant combinations is higher than the control significantly. The scavenging ability at initial state is the highest, and the scavenging rate showed a decreasing trend with walnut oxidation (Figure 4). After 4 days of oxidation, the scavenging rates of TT, TS, DS, and DT had no significance to the positive control, which indicated that these antioxidants had similar effects as positive. On day 7, the scavenging rates of TT and DS were higher than positive. Like other indicators, TT and DS showed good antioxidant capacity again. On day 9, the scavenging rate of DS was higher than other combinations significantly, and TR and DT were higher than positive significantly. The results indicated that DS can delay the oxidation process of the walnut, and the antioxidant effect of DS is better than positive significantly.

Based on the results of POV, AV, TBARS, and DPPH radical scavenging activity, the combination of DLTP and SA (referred to as DS) was identified as the optimal antioxidant combinations. DS significantly inhibited the formation of hydroperoxides and malondialdehyde during the 9-day accelerated oxidation period and maintained high DPPH radical scavenging activity in the later stages of oxidation [46]. As a fat-soluble antioxidant, DLTP antioxidant combinations offer antioxidant and stabilizing properties with low toxicity and a cost-effective profile, making it a promising candidate for widespread application. Sodium ascorbate (SA), a water-soluble derivative of ascorbic acid, is commonly used as an antioxidant and acidity regulator in fresh-cut fruits and vegetables, canned foods, meat products, and beverages [54]. It is widely used in food processing and considered to have no toxic effect on consumers [55]. The combination of DLTP and SA demonstrated a synergistic effect superior to that of commercial compound antioxidants. Therefore, DS can be considered an excellent substitute for commercial compound antioxidants.

### 3.5. Fatty Acid

UFAs in walnut kernels confer significant benefits to human cardiovascular and cerebrovascular health. Table 2 presents the relative fatty acid composition of walnut oil during oxidation. Initially, walnut oil contains 6.00% palmitic acid, 2.92% stearic acid, 20.66% oleic acid, 59.81% linoleic acid, and 10.53% linolenic acid, corresponding to 8.92% saturated fatty acids (SFA) and 91.00% UFAs. Throughout the oxidation process, SFA content increased but remained below 10.4%. Elevated SFA levels can contribute to cardiovascular diseases by increasing low-density lipoprotein cholesterol in human blood [56]. UFAs in walnut oil decreased but remained above 89.6%, attributed to the instability of the C=C bonds in fatty acids, rendering UFAs highly susceptible to oxidation [57]. Although the overall fatty acid composition of walnut oil remained consistent during accelerated oxidation, variations among groups indicated that different antioxidants exert distinct effects on delaying walnut oil oxidation. On day 4, the proportion of UFAs in TT and positive groups was similar, with no significant differences, whereas the UFA content in DS and other groups was significantly lower than in the positive control. By day 7, the UFA proportion in the TP group was significantly higher than in the positive control, while the DS, TS, and TT groups showed no significant differences compared to the positive control. On day 9, the UFA proportion in DS was significantly higher than in the positive control, while the DT, TT, BR, TP, and TR groups exhibited slightly higher UFA proportions than the positive control, though not significantly.

### 3.6. Thermogravimetric Analysis

**Figure 5:**
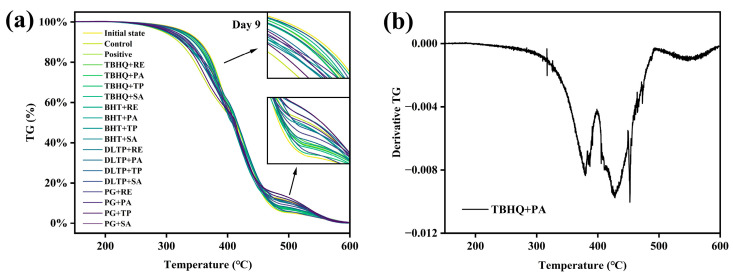
Thermal decomposition of walnut oil with different compound antioxidants: the thermogravimetric (**a**) and derivative thermogravimetric curves (**b**).

Thermogravimetric (TG) continuously monitors weight changes in walnut oil due to oxidation under programmed heating conditions, providing insights into the oil’s oxidative stability [40]. Figure 5 presents the thermogravimetric (TG) and differential thermogravimetric (DTG) curves for walnut oil. Figure 5a compares the TG curves of untreated walnut oil and walnut oil treated with various antioxidants after 9-day oxidation. The heating program reached 600 °C, resulting in complete oxidation and decomposition of walnut oil, with only 0.2% of the initial weight remaining.

To further analyze the oxidation process, the first-order derivative of the TG curve was taken to generate the DTG curve. All curves exhibited two major weight loss stages. For instance, the TP group (Figure 5b) showed the first weight loss stage between 300 °C and 400 °C, representing the primary oxidation phase of walnut oil. The second weight loss stage, between 400 °C to 500 °C, is attributed to the decomposition of the benzene ring, which has higher bond energy due to its unique structure and π-π conjugation, and thus decomposes only at higher temperatures [58]. The first weight loss stage was analyzed using baseline extrapolation. The oxidation decomposition temperature, as well as the 5% and 10% decomposition temperatures, were calculated for walnut oil (Table 3). As detailed in Table 3, the initial decomposition temperature of walnut oil was 355.54 °C, which decreased following oxidation. Compared to the control, the addition of antioxidants can effectively delay the onset temperature for thermal decomposition, a finding consistent with the experimental results reported by Ma et al. [33]. Notably, the DS, TT, and BR groups demonstrated superior antioxidant capacity to the positive control. The initial 5% decomposition temperature of walnut oil was 332.15 °C, and antioxidants consistently increased this temperature, with DS, BR, and TT outperforming the positive control. Similarly, the initial 10% decomposition temperature was 355.04 °C, with decomposition temperatures ranking in descending order as DS, TT, BR, and TP. Unlike the variable antioxidant efficacy of BR and TP, DS and TT provided comprehensive protection. During oxidation, DS and TT effectively increased the decomposition temperature, thereby safeguarding walnut oil and mitigating oxidation.

### 3.7. Electron Spin Resonance

**Figure 6:**
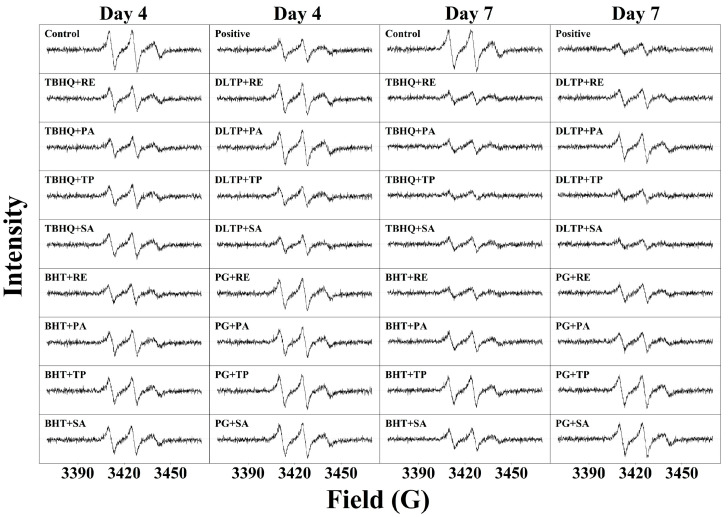
The ESR spectra of different antioxidant combinations on walnuts at days 4 and 7.

The intensity of free radical signals, indicative of lipid oxidation, can be assessed using electron spin resonance (ESR) spectroscopy [59]. In this study, ESR combined with a spin-trapping technique was employed to identify and quantify free radicals in walnut oil during the heating process, utilizing DMPO as the spin trap. As shown in Figure 6, the consistency in free radical types across samples is evident, with peak field strengths ranging from 3400 to 3450 G. Antioxidants significantly reduced the peak intensity compared to the control, likely by donating hydrogen atoms to free radicals generated during autoxidation, thereby blocking radical chain propagation and delaying oxidation [60].

Initially, free radical signal intensity was minimal but increased significantly after 4 days of oxidation, reflecting the accumulation of free radicals in walnut oil captured by DMPO. On day 4, groups with lower signal intensity included TT, DS, positive control, DT, BR, TR, TP, and TS. By day 7, DS, TP, DT, BR, positive control, TT, TR, BP, and TS exhibited superior antioxidant effects compared to the control. Corroborated by TG analysis, these results indicate that groups with fewer free radical signals corresponded to higher decomposition temperatures. In summary, TT and DS demonstrated robust antioxidant capacity, effectively delaying walnut oil oxidation throughout the process and outperforming the positive control, suggesting their potential as alternatives to commercial compound antioxidants.

## 4. Conclusions

This study systematically investigated the synergistic interactions between fat-soluble antioxidants (TBHQ, BHT, DLTP, and PG) and water-soluble antioxidants (RE, PA, TPs, and SA) in oil-in-water (O/W) emulsions for walnut preservation. The results demonstrated that specific binary antioxidant combinations 0.03% TBHQ + 0.03% TPs and 0.03% DLTP + 0.03% SA (*w*/*w*) significantly enhanced the oxidative stability of walnut kernels. These formulations effectively suppressed the increase in POV, AV, and TBARS, thereby preserving the integrity of UFAs in walnuts. Furthermore, these combinations displayed superior antioxidant performance compared to commercial blends, as evidenced by their high DPPH radical scavenging rates, reduced free radical intensity, and increased thermal decomposition temperature of walnut oil. In addition to TBHQ-TPs and DLTP-SA, other binary combinations such as TBHQ-PA, DLTP-TPs, and BHT-RE also demonstrated antioxidant capacities comparable to those of commercially available formulations. Mechanistic insights obtained through thermogravimetric analysis and electron paramagnetic resonance spectroscopy further elucidated the synergistic interactions between the components of these binary systems. These findings underscore the potential of binary antioxidant combinations for preserving lipid-rich foods like walnuts. This study offers practical solutions for extending the shelf life and maintaining the nutritional quality of walnuts.

## Figures and Tables

**Table 1:** The additive amount of fat- and water-soluble antioxidants added in the sample groups.

	Group Abbreviation	Fat-Soluble		Water-Soluble	
		Antioxidants	Amount (%, *w*/*w*)	Antioxidants	Amount (%, *w*/*w*)
1	TR	TBHQ	0.03	RE	0.045
2	TP	TBHQ	0.03	PA	0.03
3	TT	TBHQ	0.03	TPs	0.03
4	TS	TBHQ	0.03	SA	0.03
5	BR	BHT	0.03	RE	0.045
6	BP	BHT	0.03	PA	0.03
7	BT	BHT	0.03	TPs	0.03
8	BS	BHT	0.03	SA	0.03
9	DR	DLTP	0.03	RE	0.045
10	DP	DLTP	0.03	PA	0.03
11	DT	DLTP	0.03	TPs	0.03
12	DS	DLTP	0.03	SA	0.03
13	PR	PG	0.015	RE	0.045
14	PP	PG	0.015	PA	0.03
15	PT	PG	0.015	TPs	0.03
16	PS	PG	0.015	SA	0.03

TBHQ = Tert-butylhydroquinone, BHT = Butylated Hydroxytoluene, DLTP = Dilauryl Thiodipropionate, PG = Propyl Gallate, RE = Rosemary Extract, PA = Phytic Acid, TPs = Tea Polyphenols, SA = Sodium Ascorbate. The amounts of antioxidants added was calculated based on their weight ratio (*w*/*w*) relative to the O/W emulsion.

**Table 2:** The changes in fatty acid composition of walnut kernel composition during accelerated oxidation process with different antioxidant combinations.

Days of Oxidation	Group	Palmitic Acid (C16:0, %)	Stearic Acid (C18:0, %)	Oleic Acid (C18:1, %)	Linoleic Acid (C18:2, %)	α-Linolenic Acid (C18:3, %)	SFAs	UFAs
	Initial state	6.00 ± 0.01	2.92 ± 0.00	20.66 ± 0.04	59.81 ± 0.04	10.53 ± 0.01	8.92 ± 0.01	91.00 ± 0.01
Day 4	Control	6.64 ± 0.05 ^a^	3.05 ± 0.00 ^d^	17.96 ± 0.01 ^j^	61.51 ± 0.03 ^f^	10.76 ± 0.00 ^h^	9.69 ± 0.04 ^a^	90.23 ± 0.04 ^j^
	Positive	6.38 ± 0.01 ^cde^	2.62 ± 0.01 ^k^	17.31 ± 0.01 ^m^	62.05 ± 0.04 ^d^	11.56 ± 0.04 ^b^	9.01 ± 0.00 ^ij^	90.92 ± 0.00 ^ab^
	TR	6.25 ± 0.03 ^fg^	2.92 ± 0.00 ^g^	19.02 ± 0.01 ^d^	61.07 ± 0.05 ^i^	10.65 ± 0.02 ^i^	9.18 ± 0.03 ^fg^	90.74 ± 0.04 ^de^
	TP	6.05 ± 0.03 ^h^	3.06 ± 0.00 ^cd^	18.25 ± 0.01 ^g^	61.21 ± 0.02 ^h^	11.35 ± 0.03 ^e^	9.11 ± 0.03 ^gh^	90.81 ± 0.02 ^cd^
	TT	6.19 ± 0.01 ^g^	2.85 ± 0.01 ^i^	19.94 ± 0.02 ^a^	60.66 ± 0.03 ^k^	10.27 ± 0.00 ^k^	9.05 ± 0.02 ^hi^	90.87 ± 0.02 ^bc^
	TS	6.33 ± 0.03 ^ef^	2.86 ± 0.01 ^i^	17.03 ± 0.02 ^n^	62.66 ± 0.00 ^b^	11.05 ± 0.01 ^g^	9.18 ± 0.03 ^efg^	90.74 ± 0.03 ^de^
	BR	6.24 ± 0.04 ^fg^	2.89 ± 0.01 ^h^	18.41 ± 0.00 ^f^	60.82 ± 0.05 ^j^	11.56 ± 0.02 ^b^	9.13 ± 0.04 ^gh^	90.78 ± 0.04 ^cd^
	BP	6.27 ± 0.04 ^fg^	3.05 ± 0.01 ^d^	18.08 ± 0.03 ^i^	61.42 ± 0.02 ^fg^	11.11 ± 0.03 ^f^	9.32 ± 0.04 ^cd^	90.60 ± 0.04 ^gh^
	BT	6.17 ± 0.01 ^g^	3.07 ± 0.01 ^bc^	18.16 ± 0.00 ^h^	61.49 ± 0.03 ^f^	11.02 ± 0.01 ^g^	9.25 ± 0.02 ^def^	90.67 ± 0.02 ^efg^
	BS	6.39 ± 0.03 ^cde^	2.98 ± 0.00 ^f^	17.27 ± 0.02 ^m^	61.44 ± 0.02 ^fg^	11.84 ± 0.01 ^a^	9.37 ± 0.03 ^c^	90.55 ± 0.03 ^h^
	DR	6.48 ± 0.05 ^bc^	2.86 ± 0.01 ^i^	17.54 ± 0.02 ^k^	62.01 ± 0.05 ^d^	11.03 ± 0.02 ^g^	9.34 ± 0.05 ^cd^	90.58 ± 0.06 ^gh^
	DP	6.22 ± 0.05 ^g^	2.97 ± 0.01 ^f^	16.09 ± 0.02 ^o^	63.29 ± 0.03 ^a^	11.36 ± 0.02 ^de^	9.19 ± 0.05 ^efg^	90.73 ± 0.04 ^def^
	DT	6.02 ± 0.04 ^h^	3.17 ± 0.01 ^a^	19.14 ± 0.02 ^c^	60.01 ± 0.02 ^m^	11.58 ± 0.01 ^b^	9.19 ± 0.04 ^efg^	90.73 ± 0.04 ^def^
	DS	6.45 ± 0.01 ^cd^	2.65 ± 0.01 ^j^	18.61 ± 0.01 ^e^	61.76 ± 0.01 ^e^	10.47 ± 0.00 ^j^	9.10 ± 0.02 ^ghi^	90.83 ± 0.02 ^bcd^
	PR	6.27 ± 0.01 ^fg^	3.01 ± 0.01 ^e^	18.23 ± 0.02 ^g^	61.36 ± 0.01 ^g^	11.05 ± 0.01 ^g^	9.28 ± 0.02 ^cde^	90.64 ± 0.03 ^fgh^
	PP	6.38 ± 0.05 ^cde^	3.09 ± 0.00 ^b^	19.51 ± 0.03 ^b^	60.21 ± 0.02 ^l^	10.72 ± 0.01 ^h^	9.47 ± 0.05 ^b^	90.44 ± 0.05 ^i^
	PT	6.35 ± 0.04 ^def^	2.94 ± 0.00 ^g^	16.99 ± 0.02 ^n^	62.24 ± 0.04 ^c^	11.41 ± 0.02 ^d^	9.28 ± 0.04 ^cde^	90.64 ± 0.03 ^fgh^
	PS	6.56 ± 0.02 ^ab^	2.98 ± 0.01 ^f^	17.45 ± 0.02 ^l^	61.44 ± 0.04 ^fg^	11.48 ± 0.02 ^c^	9.54 ± 0.02 ^b^	90.38 ± 0.01 ^i^
Day 7	Control	7.01 ± 0.02 ^a^	2.95 ± 0.02 ^c^	17.47 ± 0.01 ^o^	61.67 ± 0.04 ^b^	10.91 ± 0.01 ^n^	9.96 ± 0.04 ^a^	90.04 ± 0.04 ^l^
	Positive	6.42 ± 0.04 ^hi^	2.81 ± 0.02 ^g^	19.30 ± 0.03 ^g^	60.18 ± 0.04 ^h^	11.28 ± 0.03 ^k^	9.23 ± 0.02 ^ijk^	90.77 ± 0.02 ^bcd^
	TR	6.43 ± 0.01 ^hi^	2.87 ± 0.01 ^de^	19.55 ± 0.03 ^e^	59.45 ± 0.03 ^j^	11.71 ± 0.01 ^ef^	9.29 ± 0.01 ^ghi^	90.71 ± 0.01 ^def^
	TP	6.37 ± 0.01 ^i^	2.80 ± 0.01 ^g^	19.40 ± 0.01 ^f^	60.40 ± 0.01 ^f^	11.03 ± 0.01 ^m^	9.17 ± 0.02 ^kl^	90.83 ± 0.02 ^ab^
	TT	6.46 ± 0.05 ^gh^	2.82 ± 0.01 ^fg^	21.67 ± 0.04 ^a^	57.84 ± 0.03 ^m^	11.22 ± 0.02 ^l^	9.27 ± 0.05 ^hij^	90.73 ± 0.05 ^cde^
	TS	6.25 ± 0.03 ^j^	2.97 ± 0.01 ^c^	20.69 ± 0.01 ^b^	57.97 ± 0.03 ^l^	12.13 ± 0.02 ^a^	9.21 ± 0.02 ^ijk^	90.79 ± 0.02 ^bcd^
	BR	6.53 ± 0.03 ^fg^	2.84 ± 0.00 ^efg^	19.66 ± 0.03 ^d^	59.77 ± 0.00 ^i^	11.21 ± 0.03 ^l^	9.37 ± 0.03 ^fg^	90.63 ± 0.03 ^fg^
	BP	6.63 ± 0.05 ^de^	2.99 ± 0.01 ^c^	17.45 ± 0.02 ^o^	61.16 ± 0.02 ^d^	11.77 ± 0.02 ^d^	9.62 ± 0.06 ^cde^	90.38 ± 0.06 ^hij^
	BT	6.75 ± 0.00 ^bc^	2.86 ± 0.01 ^def^	18.26 ± 0.01 ^l^	60.91 ± 0.01 ^e^	11.22 ± 0.02 ^l^	9.61 ± 0.01 ^de^	90.39 ± 0.01 ^hi^
	BS	6.76 ± 0.00 ^b^	2.89 ± 0.03 ^d^	16.80 ± 0.02 ^p^	61.53 ± 0.03 ^c^	12.02 ± 0.01 ^b^	9.65 ± 0.03 ^bcd^	90.35 ± 0.03 ^ijk^
	DR	6.67 ± 0.03 ^cd^	3.04 ± 0.00 ^b^	16.62 ± 0.01 ^q^	62.07 ± 0.03 ^a^	11.60 ± 0.01 ^g^	9.71 ± 0.03 ^bc^	90.29 ± 0.03 ^jk^
	DP	6.42 ± 0.02 ^hi^	2.88 ± 0.01 ^de^	19.05 ± 0.02 ^i^	60.17 ± 0.01 ^h^	11.48 ± 0.02 ^ij^	9.29 ± 0.01 ^ghi^	90.71 ± 0.01 ^def^
	DT	6.59 ± 0.00 ^def^	2.75 ± 0.01 ^h^	19.73 ± 0.01 ^c^	59.25 ± 0.04 ^k^	11.68 ± 0.02 ^f^	9.34 ± 0.02 ^gh^	90.66 ± 0.02 ^ef^
	DS	6.57 ± 0.03 ^ef^	2.62 ± 0.01 ^i^	18.97 ± 0.01 ^j^	60.28 ± 0.04 ^g^	11.56 ± 0.01 ^gh^	9.19 ± 0.04 ^jkl^	90.81 ± 0.04 ^abc^
	PR	6.66 ± 0.00 ^d^	2.89 ± 0.04 ^d^	18.41 ± 0.03 ^k^	60.15 ± 0.05 ^h^	11.89 ± 0.00 ^c^	9.55 ± 0.04 ^e^	90.45 ± 0.04 ^h^
	PP	6.46 ± 0.02 ^gh^	2.98 ± 0.00 ^c^	19.17 ± 0.04 ^h^	59.42 ± 0.04 ^j^	11.97 ± 0.01 ^d^	9.44 ± 0.02 ^f^	90.56 ± 0.02 ^g^
	PT	6.83 ± 0.04 ^b^	2.81 ± 0.00 ^fg^	17.64 ± 0.02 ^n^	61.20 ± 0.03 ^d^	11.52 ± 0.01 ^hi^	9.64 ± 0.04 ^bcde^	90.36 ± 0.04 ^hijk^
	PS	6.60 ± 0.03 ^def^	3.11 ± 0.01 ^a^	17.71 ± 0.03 ^m^	61.11 ± 0.01 ^d^	11.47 ± 0.02 ^j^	9.71 ± 0.03 ^b^	90.29 ± 0.03 ^k^
Day 9	Control	7.31 ± 0.02 ^a^	2.84 ± 0.00 ^de^	17.23 ± 0.01 ^h^	60.16 ± 0.02 ^f^	12.36 ± 0.01 ^abc^	10.16 ± 0.02 ^a^	89.76 ± 0.02 ^g^
	Positive	6.72 ± 0.02 ^ij^	2.97 ± 0.01 ^a^	19.08 ± 0.05 ^b^	58.97 ± 0.09 ^j^	12.18 ± 0.01 ^de^	9.68 ± 0.03 ^defg^	90.23 ± 0.03 ^abcd^
	TR	6.76 ± 0.03 ^hij^	2.93 ± 0.02 ^bc^	16.35 ± 0.04 ^l^	61.89 ± 0.10 ^a^	11.99 ± 0.19 ^fg^	9.68 ± 0.05 ^defg^	90.23 ± 0.05 ^abcd^
	TP	6.97 ± 0.08 ^def^	2.70 ± 0.01 ^i^	16.87 ± 0.03 ^j^	60.94 ± 0.10 ^d^	12.44 ± 0.02 ^ab^	9.67 ± 0.07 ^defg^	90.25 ± 0.07 ^abcd^
	TT	6.69 ± 0.02 ^j^	2.92 ± 0.00 ^bc^	18.44 ± 0.04 ^d^	59.88 ± 0.07 ^g^	11.98 ± 0.03 ^fg^	9.62 ± 0.02 ^efg^	90.29 ± 0.03 ^abc^
	TS	6.91 ± 0.02 ^efg^	2.82 ± 0.01 ^e^	18.48 ± 0.00 ^d^	59.34 ± 0.04 ^hi^	12.37 ± 0.02 ^abc^	9.73 ± 0.03 ^def^	90.18 ± 0.03 ^bcd^
	BR	6.90 ± 0.04 ^efg^	2.76 ± 0.01 ^fg^	16.20 ± 0.04 ^m^	61.59 ± 0.05 ^b^	12.46 ± 0.03 ^a^	9.66 ± 0.04 ^defg^	90.25 ± 0.05 ^abcd^
	BP	7.01 ± 0.03 ^cde^	2.71 ± 0.01 ^hi^	16.96 ± 0.03 ^j^	60.95 ± 0.05 ^d^	12.30 ± 0.02 ^bcd^	9.71 ± 0.04 ^def^	90.21 ± 0.04 ^bcd^
	BT	7.11 ± 0.03 ^bc^	2.90 ± 0.01 ^c^	16.52 ± 0.01 ^k^	61.44 ± 0.04 ^bc^	11.94 ± 0.01 ^fg^	10.01 ± 0.03 ^b^	89.90 ± 0.03 ^f^
	BS	7.01 ± 0.04 ^cde^	2.75 ± 0.00 ^fg^	19.20 ± 0.02 ^a^	59.32 ± 0.02 ^i^	11.63 ± 0.02 ^h^	9.77 ± 0.04 ^cd^	90.15 ± 0.04 ^de^
	DR	6.80 ± 0.03 ^ghij^	2.95 ± 0.01 ^ab^	17.54 ± 0.00 ^f^	61.37 ± 0.02 ^c^	11.26 ± 0.04 ^i^	9.75 ± 0.04 ^de^	90.17 ± 0.03 ^cd^
	DP	7.15 ± 0.06 ^b^	2.84 ± 0.02 ^de^	17.24 ± 0.02 ^h^	60.33 ± 0.03 ^ef^	12.36 ± 0.03 ^abc^	9.99 ± 0.05 ^b^	89.93 ± 0.05 ^f^
	DT	6.84 ± 0.06 ^ghi^	2.78 ± 0.00 ^f^	17.42 ± 0.01 ^g^	61.59 ± 0.04 ^b^	11.29 ± 0.03 ^i^	9.62 ± 0.06 ^fg^	90.30 ± 0.06 ^ab^
	DS	6.86 ± 0.03 ^fgh^	2.70 ± 0.01 ^hi^	18.80 ± 0.01 ^c^	59.50 ± 0.04 ^h^	12.05 ± 0.02 ^ef^	9.57 ± 0.03 ^g^	90.35 ± 0.03 ^a^
	PR	6.99 ± 0.05 ^cdef^	2.78 ± 0.01 ^f^	17.12 ± 0.04 ^i^	60.81 ± 0.03 ^d^	12.23 ± 0.01 ^cd^	9.76 ± 0.04 ^cd^	90.15 ± 0.04 ^de^
	PP	7.07 ± 0.01 ^bcd^	2.86 ± 0.01 ^d^	17.81 ± 0.06 ^e^	60.22 ± 0.08 ^ef^	11.96 ± 0.03 ^fg^	9.93 ± 0.01 ^b^	89.98 ± 0.01 ^f^
	PT	7.18 ± 0.03 ^ab^	2.74 ± 0.02 ^gh^	17.25 ± 0.01 ^h^	60.36 ± 0.03 ^e^	12.38 ± 0.03 ^abc^	9.92 ± 0.03 ^b^	89.99 ± 0.03 ^f^
	PS	7.06 ± 0.01 ^bcd^	2.83 ± 0.02 ^de^	17.29 ± 0.01 ^h^	60.80 ± 0.02 ^d^	11.94 ± 0.02 ^fg^	9.89 ± 0.01 ^bc^	90.03 ± 0.01 ^ef^

The results (%) are expressed as the ratio of the individual fatty acid content to the total fatty acid content. SFAs = Saturated Fatty Acids, UFAs = Unsaturated Fatty Acids. Letters indicate significant differences among different groups in the same column by Tukey’s test (*p* < 0.05). Values are presented as mean ± SD of triplicates.

**Table 3:** The oxidation decomposition temperature of walnut with different antioxidant combinations after 9-day oxidation.

Groups	Oxidation Decomposition Temperature/(°C)	5% Oxidation Decomposition Temperature/(°C)	10% Oxidation Decomposition Temperature/(°C)
Initial State	355.54	332.15	355.04
Control	308.78	294.69	319.80
Positive	346.12	326.90	349.14
TR	341.98	320.97	345.45
TP	345.17	325.25	349.92
TT	353.47	327.76	353.06
TS	343.09	321.05	345.05
BR	352.51	328.26	351.78
BP	341.97	298.08	326.95
BT	329.53	302.34	329.61
BS	324.26	309.96	331.97
DR	326.33	299.54	324.93
DP	336.87	316.10	338.38
DT	344.05	322.50	346.33
DS	354.58	329.42	353.38
PR	327.45	309.56	335.05
PP	338.94	307.74	332.22
PT	329.53	313.74	337.54
PS	318.03	303.84	326.74

## Data Availability

The original contributions presented in this study are included in the article. Further inquiries can be directed to the corresponding author.

## Abbreviations

The following abbreviations are used in this manuscript:

UFAs	Unsaturated fatty acids
POV	Peroxide value
AV	Acid value
TBARS	Thiobarbituric acid reactive substances
DPPH	1,1-Diphenyl-2-picrylhydrazyl radical
EPA	Eicosapentaenoic acid
DHA	Docosahexaenoic acid
FA	Fatty acid composition
TG	Thermogravimetric analysis
ESR	Electron spin resonance
O/W	Oil in water
FAMEs	Fatty acid methyl esters
